# Neurophysiological Modulations of Non-Verbal and Verbal Dual-Tasks Interference during Word Planning

**DOI:** 10.1371/journal.pone.0168358

**Published:** 2016-12-19

**Authors:** Raphaël Fargier, Marina Laganaro

**Affiliations:** Faculty of Psychology and Educational Sciences, University of Geneva, Geneva, Switzerland; University of Akron, UNITED STATES

## Abstract

Running a concurrent task while speaking clearly interferes with speech planning, but whether verbal vs. non-verbal tasks interfere with the same processes is virtually unknown. We investigated the neural dynamics of dual-task interference on word production using event-related potentials (ERPs) with either tones or syllables as concurrent stimuli. Participants produced words from pictures in three conditions: without distractors, while passively listening to distractors and during a distractor detection task. Production latencies increased for tasks with higher attentional demand and were longer for syllables relative to tones. ERP analyses revealed common modulations by dual-task for verbal and non-verbal stimuli around 240 ms, likely corresponding to lexical selection. Modulations starting around 350 ms prior to vocal onset were only observed when verbal stimuli were involved. These later modulations, likely reflecting interference with phonological-phonetic encoding, were observed only when overlap between tasks was maximal and the same underlying neural circuits were engaged (cross-talk).

## Introduction

We can produce more than one hundred words per minute with such a great accuracy that one would assume speaking is a highly automatic process. However, the fact that language production requires at least some degree of attention is illustrated by the effort incurred by speakers to maintain a fluent conversation while performing a concurrent task (e.g. talking while driving in heavy traffic) [1]. It is however likely that the double task does not affect all speech encoding processes to the same extent, as some processes might require increased attentional demand while others may be more automatic.

There is a general agreement on the various processing stages occurring during word production. These processes include prelinguistic or semantic encoding, lexical selection, phonological word form retrieval and encoding and postlexical phonetic and pre-articulatory programming [2,3]. Whether these processes are strictly distinct and ordered serially [2,4,5] or partially overlap [3] is among the properties that distinguish the various models of lexical access, whereas the degree of automaticity of speech planning processes is much underspecified in all models. A first insight into the capacity demand of speech planning came from studies that used gaze tracking to look at attention orientation during language production in multiple picture naming tasks [6–11]. These studies indicated that participants seem to keep their gaze at target objects until they have achieved phonological word form retrieval but shift their gaze afterwards. This is supported by the fact that gaze durations on to-be-named pictures are affected by phonological priming [7] or by phonological factors such as word length [6]. The observation that participants shift their gaze before speech onset has been interpreted as an index that post-lexical and pre-articulatory processes do not need the same amount of attention as lexical and pre-lexical processes. This is coherent with the idea that response selection is under controlled attention whereas response execution is automatic [12,13].

Dual-task paradigms were also carried out to test the attention requirements in speech production [10]. These studies were conducted under the assumption that the central attention demands of a given cognitive process are indexed by the extent to which this process affects (i.e. delays or speeds) the performance of an unrelated concurrent task [14]. The psychological refractory period (PRP) paradigm, in particular, has been largely used to this aim [15–17]. In the PRP paradigm, two distinct targets (T1 and T2) are presented in a sequential order, and participants have to give a speeded response for each target. Many studies reported interference effects illustrated by increase in response time to the second target as the interval between the two targets decreases (Stimulus Onset Asynchrony, SOA) [18]. In their seminal study on dual-task in speech production, Ferreira & Pashler (2002) [19] had participants undergoing a picture-naming task (Task1) while concurrently performing an auditory tone discrimination task (Task2). They manipulated the ease of lexical selection on one hand (using high versus low cloze constraints of the written sentence preceding the picture to be named) and of phonological word form selection on the other hand (using pictures corresponding to high versus low lexical frequency words). They found that similar to the naming latencies, tone discrimination latencies were slower when pictures were named in the low-constraint cloze sentence condition. Moreover, in this latter condition, responses to tones were slower when presented after pictures with low frequency names. The authors argued that these effects occurred because the resolution of Task2 could not begin at least until both lexical selection and phonological word-form retrieval (of Task1) were completed. The data obtained by Ferreira & Pashler (2002) thus imply that both lexical selection and phonological word-form encoding are under attention demand. In a subsequent experiment, Task1 was replaced by the picture-word interference paradigm, with either category-related word distractors or phonologically-related ones. Tone-discrimination latencies were modulated only for category-related distractors [19], which led Ferreira & Pashler (2002) to argue that lexical selection was under attention demand but that phonological encoding was not (see also [10] for related observations). Yet, Cook & Meyer (2008) [20] found that facilitatory effects of phonologically related distractors on picture naming propagated to tone discrimination latencies, but only when distractors were pictures or masked words. The authors assumed that visible distractors (as in Ferreira & Pashler’s study) would induce a facilitation of phoneme selection, but that this effect might be counterbalanced by slowed down speech monitoring processes occuring in the meantime. Accordingly, whereas PRP and eye tracking experiments converge to the idea that lexical selection is under attentional demand, the picture is far less clear for phonological encoding and speech-motor programming. It is possible that the degree of attentional demand might depend on cross-talk mechanisms [21,22] i.e. on the degree of overlap between the tasks. One way to manipulate this could thus be to use verbal and non-verbal tasks as concurrent tasks.

In the present study, we sought to examine the attention requirements of word encoding stages using event-related potentials (ERPs) during a dual tasks paradigm. Recent advances in ERP methodology have allowed the study of overt word production with EEG [23] including taking into account the variability in word production latencies across experimental conditions. Furthermore, spatio-temporal segmentation analyses can reveal differences in the configurations of the scalp topography [24] therefore making possible to delineate the time-windows that likely correspond to specific mental processes, and examine whether they are shortened or extended as a function of experimental conditions. This approach has been successful to determine the locus of age-of-acquisition effects in picture naming [25,26] as well as showing that between-subjects speed variability is accounted for by differences around 200–350 ms after picture onset, likely associated to lexical selection [27,28].

We therefore took advantage of this approach to address i) which speech planning time-windows, i.e. which word encoding processes are under attentional demand and ii) whether the attention requirements of specific encoding stages are sensitive to the verbal/non-verbal nature of the concurrent task.

For this, we asked participants to perform three tasks that varied according to the degree of involved attentional demand: Picture naming only (single task or ST), picture naming while ignoring auditory stimuli (passive dual-task or pDT) and picture naming during an auditory stimulus detection task (active dual-task or aDT). These experimental tasks were conducted with either tones or syllables to explore potential differences due to the linguistic nature of distractors (non verbal versus verbal distractor).

In all concurrent tasks, auditory stimuli appeared 300 ms after the onset of the picture on the screen (SOA = +300 ms). In the passive dual-task, participants were requested to name the pictures while ignoring the distractors. For the sake of ERPs, in the auditory distractor detection task or active dual-task, we used a go/no go paradigm so that participants were asked to respond on a button box when they heard one specified tone (or syllable) (filler items) while continuing to name all the pictures. This allowed us to directly compare word production between the three tasks. The SOA was chosen to directly tap into lexical processes likely occuring between 200–400 ms post picture onset (see Indefrey, 2011 for a meta-analysis). This allowed us to examine to what extent this encoding stage and the next ones were affected by the concurrent tasks.

## Material and Methods

### Participants

22 undergraduate students (9 men) took part in the study. All were native French speakers (mean age = 23.7 ± 7.8 years, age range 19–52 years) and right-handed as determined by the Edinburgh Handedness Scale [29]. Only 17 subjects out of 22 were selected for further analysis (n.b. for tones; when distractors were syllables 18 subjects were kept) due to bad EEG signal and/or insufficient number of epochs for at least one of the conditions (see Results section).

None of the participants had a history of psychiatric or neurological disorders and had normal or corrected-to-normal vision. They gave their informed written consent in accordance with the declaration of Helsinki (1968) and got credit courses for their participation. Procedures were approved by the ethics committee of Geneva, Switzerland.

### Stimuli

216 pictures (line drawings on white squares of 240 x 245 pixels) and their corresponding words were used as target pictures. Pictures depicted objects and were selected from two French databases [30,31] and all had high name agreement (>80%). Pictures were divided into three matched lists of items to be used for the three tasks: single task (ST), passive dual-task (pDT) and active dual-task (aDT). Psycholinguistic variables associated to the pictures and the corresponding words were matched across lists: Name agreement, image agreement, familiarity, subjective visual complexity, image variability and age-of-acquisition (from the mentioned databases), lexical frequency, phonological neighborhood density, length in phonemes (from Lexique, [32]) but also on sonority of the first phoneme on an 8-points French sonority scale (all F<1). Pictures were also matched for objective visual complexity defined as the perimeter of line drawings assessed through Canny perimeter-detection calculation.

36 additional pictures and the corresponding nouns were selected from the same databases as filler items, to be associated with the target distractors (excluded from the analyses) and 12 pictures were added as warming-up/training filler trials.

For the tones dual-tasks, we selected five different tones varying in pitches (lowest (180 Hz), half-low (450 Hz), medium (700 Hz), half-high (900 Hz), highest (1200 Hz)). Considering the variations in pitches, it appeared that the lowest pitch was the easiest to detect and was therefore chosen as “target” distractor in the picture naming with detection of auditory (tone) distractor. We also selected five different syllables (/ri/, /na/, /mi/, /de/, /fo/) following the criterion that there was no phonological overlap between the distractor syllable and the first syllable of the target word. The syllable /fo/ was chosen as “target” distractor in the picture naming with detection of auditory (syllable) distractor. The duration of auditory stimuli was 280 ms for both syllables and tones. There were 72 trials per task (+18 filler “target” items in dual-tasks). All pictures were repeated twice, once for the tasks with tones as auditory distractors, and once for the tasks with syllables as auditory distractors. Lists of items were counterbalanced across subjects.

### Procedure

Participants were tested individually in a soundproof dark room. The presentation of trials was controlled by the E-Prime software (E-Studio). Pictures were presented in constant size of 240 x 245 pixels (about 4.52° of visual angle) on a black screen (approximately 60 cm from their chest).

The tasks were run in separate blocks in which all items were presented in a pseudo-random order and were preceded by 4 warming-up filler trials. The order of blocks was counter-balanced across subjects. Within subjects, the order of blocks was constant across distractors such that when the experiments started with the active dual-task for tones for instance, it was followed by the active dual-task for syllables.

The three different tasks were as follows:

In the single task (ST), an experimental trial began with a fixation cross presented for 500 ms. Then the picture appeared on the screen for 2000 ms. Participants were requested to produce overtly the word corresponding to the picture as fast and accurate as possible. A blank screen lasting 2000 ms was displayed before the next trial.

In the passive dual-task (pDT), the experimental trial was similar except that an auditory distractor appeared 300 ms after the onset of the picture (SOA = +300 ms). Participants were requested to produce overtly the word corresponding to the picture as soon as they could while ignoring the distractor. Auditory distractors could be tones or syllables (in separate blocks).

In the active dual-task (aDT), the experimental trials were similar as in the pDT except that participants were requested to detect one particular target distractor by pressing a button on a button box while concurrently producing overtly the word corresponding to the picture. They were asked to respond to both tasks as soon and accurate as they could.

In both active dual-tasks, target stimuli corresponded to 20% of the items (associated to the 18 filler items and excluded from the analyses). Hence, the go response was only on filler items, whereas no manual response was required on the target items which were compared across tasks/conditions.

For the dual-task in which the distractors were tones, the instruction was given to press a button on a response box for tones with the lowest pitch. Before this task, participants were briefly trained with the various tones and were given feedback if they failed to detect the correct target tone. For the dual-task in which the distractors were syllables, the instruction was given to press the button on the response box for the syllable /fo/. The same feedback was given in a brief training. Each block lasted about 5–8 min with a break after each block.

### EEG Acquisition and Pre-analyses

EEG was recorded continuously using the Active-Two Biosemi EEG system (Biosemi V.O.F. Amsterdam, The Netherlands) with 128 electrodes covering the entire scalp. Signals were sampled at 512 Hz (filters: DC to 104 Hz, 3 dB/octave slope).

EEG activity was analyzed using the Cartool software [33]. Stimulus-aligned (600 ms) and response-aligned epochs (500 ms) were averaged for each task. Stimulus-aligned epochs started 100 ms before the onset of the picture to 500 ms post picture onset whereas response-aligned epochs were locked to the individual production latency of each trial (100 ms before vocal onset). For the spatio-temporal analysis, in order to cover the exact time period corresponding to the mean RT for each condition, the time frames corresponding to the overlapping signal were removed from the response-aligned grand averages.

Epochs in which amplitudes exceeded ±100 μV were rejected. In addition to this automated criterion, each trial was visually inspected and epochs contaminated by eye blinking, movement artefacts or other noise were excluded. Only trials corresponding to correct production and for which both stimulus-aligned and response-aligned epochs were available were retained for averaging. This resulted in a minimum of 31 averaged trials per subject and condition (i.e. 40% of the data was kept; mean number of epochs = 53). ERPs were bandpass-filtered to 0.2–30 Hz (2^nd^ order acausal Butterworth filter with*−*12 dB/octave roll-off) and were recalculated against the average reference.

### Behavioral analyses

Production latencies were recorded by a microphone and were digitized for further systematic latency and accuracy check with a speech analysis software (CheckVocal 2.2.6, [34]). No-responses, wrong responses (i.e. the participant produced a different name than the one expected for the picture), hesitations and/or auto-corrections during articulation were considered as errors. Behavioral data were analyzed by means of mixed-effects regression models [35–37], performed with the statistical software R (R Development Core Team, 2007) and the package lme4 [38] and lmerTest package, version 2.0–29 [39] using Satterthwaite approximation for degrees of freedom for the F statistics.

### ERP analyses

Waveform analyses as well as spatio-temporal segmentation were conducted on the grand-averages from each task and statistical analyses were performed on single-subject averages.

#### Waveform analyses

For this first analysis of waveforms, ANOVAs were computed on amplitudes of the evoked response potentials at each electrode and time point (2ms) over the stimulus-aligned and response-aligned period with the three-leveled within-subject factor of *Task* (“ST” vs. “pDT” vs. “aDT”) separately for tones and syllables (see Results section). To correct for multiple comparisons, a spatio-temporal clustering criterion was used: only differences observed over at least 5 adjacent electrodes and extending over at least 20 ms were retained with a conservative alpha criterion of 0.01 [40–42].

Statistical analyses were performed using the STEN toolbox developed by Jean-François Knebel (http://www.unil.ch/line/home/menuinst/ianbout-the-line/software--analysis-tools.html).

#### Spatio-temporal analysis

The second analysis was a topographic-pattern analysis. This method allows summarizing ERP data into a limited number of stable topographic map configurations [43]. This method is independent from the used reference electrode [44,45] and not sensitive to pure amplitude modulations across conditions: topographies of normalized maps are compared.

The segmentation was obtained with the software Ragu [46]. The main advantage of the present approach [47] over previously used approaches [33] is that the identification of the topographic patterns and the fitting procedure are performed on the same data. Hence, the similarity between the topographic maps obtained from the segmentation and the topographic maps these segmentations are assigned to during the fitting procedure is maximized, leading to increased statistical power.

The optimal number of topographic map templates that best explained the group-averaged datasets was determined using the following cross-validation procedure which was applied 50 times: Each time randomly splitting the 17 subjects (for tones; 18 subjects for syllables; see Results section) into training and test datasets of 8 subjects each and testing between 1 and 20 microstates classes. Every microstate identification run used the traditional *k*-means cluster algorithm (50 random initializations each). Statistical smoothing was used to eliminate temporally isolated maps with low strength (10 points smoothing with a penalty factor of 3). Correlations between the maps obtained in the training dataset and the group-average ERPs from the test group are computed. The number of topographic maps with the higher correlation value is retained as the optimal number of maps for the current dataset. The spatio-temporal segmentation was applied to the three grand-average data corresponding to the three tasks: Single task (ST), passive dual-task (pDT) and active dual-task (aDT). The statistical validation of this analysis was tested thanks to a microstate fitting procedure during which each time point is labeled according to the map with which it best correlated spatially. This is achieved through randomization procedures such that for each participant, the ERPs corresponding to the conditions being compared are randomly assigned to arbitrarily defined groups. ERPs in these different groups are averaged and the variables of interest are computed (here map duration and offset) for the three tasks (ST, pDT and aDT). After many repetitions, the variables of interest observed in the test dataset are compared with the empirical distributions under the null hypothesis. Details of this procedure can also be found in [47].

## Results

### Behavioral results

After removal of incorrect responses and production latencies beyond 2.5 standard deviations (11.1% of the data) as well as trials excluded for bad EEG signal (14.7% of the data) with exclusion of subjects for which less than 40% of trials were kept, the behavioral analyses were performed on 17 subjects for the tones experiment and on 18 subjects for the syllables experiment (there were 16 subjects in common in the two experiments).

For statistical analyses, mixed-effects regression models (generalized-mixed models) were conducted, which allowed the two experiments to be analyzed together. Task (ST, pDT and aDT), Distractor (tones, syllables) and Block order (1,2,3), as well as their interactions, were entered as fixed factors; for the random part of the model, the most complex random structure included task, distractor and their interaction in the random by-subject structure and random intercepts for items. This model was kept after comparison against models with simpler random structure [48].

The behavioural results are summarized in Table 1.

**Table 1:** Mean production latencies (ms) expressed as a function of Tasks and Distractors.

	*Production latencies (ms)*		
	*ST*	*pDT*	*aDT*
**Tones**	817 (SD 110)	844 (SD 117)	887 (SD 101)
**Syllables**	831 (SD 158)	870 (SD 176)	953 (SD 172)

A significant effect of Task was observed (F(2,15.7) = 18.6, p<0.001) such that naming latencies were slower in the dual-tasks in comparison to the single task. There was no main effect of Distractor (F(1,12.2) = 1.3, p = 0.27) or Block order (F(1,20.2) = 2.2, p = 0.15) but a significant interaction between Distractor and Task was observed (F(2,10.6) = 4.9, p = 0.031), with longer latencies for syllables than for tones in particular in the aDT. We therefore conducted separately the analyses for verbal and non-verbal distractors.

The models included Task (ST, pDT and aDT) and Block order (1,2,3) as well as their interactions as fixed factors and Task in the random by-subject structure and random intercepts for items.

The model applied when distractors were tones (17 subjects) indicated a significant main effect of Task (F(2,13.2) = 11.1, p = 0.0015) on production latencies. RTs corresponding to aDT were longer than in ST (t(14.4) = -4.7, p<0.001; β = -70.7, SE = 15.1) and pDT (t(13.6) = -2.35, p = 0.034; β = -41.6, SE = 17.7). The difference between pDT and ST did not reach significance (t(13.9) = -1.9, p = 0.08; β = -29.1, SE = 15.2).

The same model applied when distractors were syllables (18 subjects) indicated a significant main effect of Task (F(2,16.6 = 18.6, p<0.0001) and of block order (F(1,24.2 = 4.3, p = 0.05) such that naming latencies were slower in the first block than in the second and third blocks. RTs corresponding to aDT were longer as compared to ST (t(16.4) = -4.02, p<0.001; β = -125.9, SE = 31.3) and to pDT (t(16.2) = -5.2, p<0.001; β = -88.6, SE = 17). The difference between pDT and ST did not reach significance (t(16.6) = -1.13, p = 0.27; β = -37.3, SE = 33.1).

### ERP results

#### Tones—Waveform analysis

**Fig 1:**
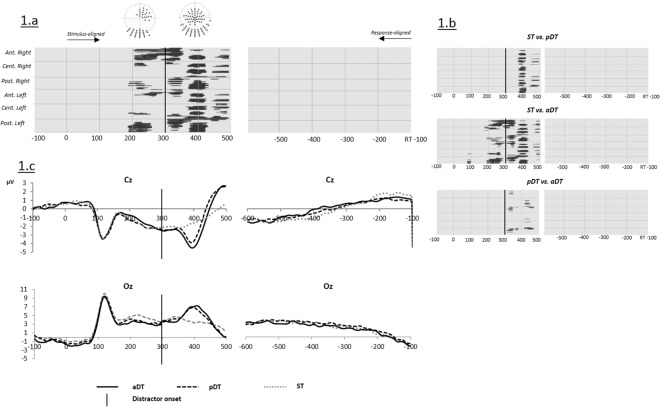
(a) Significant differences (ANOVAs p values) on ERP waveform amplitudes on each electrode (Y axis) and time point (X axis) between the three tasks (Single task ST, passive dual-task pDT and active dual-task aDT) when distractors used in dual-tasks were tones. (b) Planned comparisons of significant differences on ERP waveform amplitudes on each electrode (Y axis) and time point (X axis). (c) Example of group averaged ERP waveforms for ST (dotted gray), pDT (dotted black) and aDT (solid black) on electrodes Cz and Oz. Vertical black solid lines indicate the onset of the distractors in dual-tasks.

Fig 1 shows the time points of significant amplitude differences from picture onset to 500 ms afterwards as revealed by the ANOVA that contrasted the three tasks ST, pDT and aDT (Fig 1A) and through the planned comparisons across tasks (Fig 1B). Consistent differences were found on several time-windows from 200 ms post picture onset to 500 ms. Differences in amplitudes between 200 and 350 ms were observed mainly on right fronto-central and occipital electrodes. Here, more positive waves were observed for both dual-tasks compared to the single task on central and occipital sites.

Although still on fronto-central and occipital sites, a larger number of electrodes displayed differences between 350 and 420 ms post picture onset. These differences consisted in more negative waves for dual-tasks relative to ST on central sites and increased positivity on posterior sites. Planned comparisons (Fig 1B) revealed that the earliest differences on amplitudes (from ~200 to 350 ms) were only observed when comparing aDT to ST. Nearly exact same differences from 380 ms and 420 ms appeared on a majority of electrodes when comparing ST to pDT and ST to aDT. These effects corresponded to the presence of a positive peak in both pDT and aDT compared to ST on occipital sites and its negative counterpart on fronto-central sites (see Fig 1C). This peak that appeared between 50 and 120 ms after the onset of the distractor likely reflected the N1-P2 complex for auditory processing. Finally, modulations of amplitudes by dual-tasks as compared to single task were also found between 450 and 500 ms but restricted to central and left-lateralized temporal electrodes. The comparison across the two dual-tasks yielded differences on smaller clusters of electrodes around 320–350 ms and from 420 to 500 ms post-stimulus onset.

No significant differences on amplitudes were observed on response-aligned ERPs.

#### Tones—Spatio-temporal analysis

A spatio-temporal segmentation analysis was run separately on the stimulus-aligned and the response-aligned grand-averaged ERPs of the three tasks associated with tones. We found 9 different periods of quasi-stable electrophysiological activity at scalp on the stimulus-aligned data and 4 topographic maps on the response-aligned data. The same sequence of topographic pattern was observed in all conditions but with different time-distribution.

**Fig 2:**
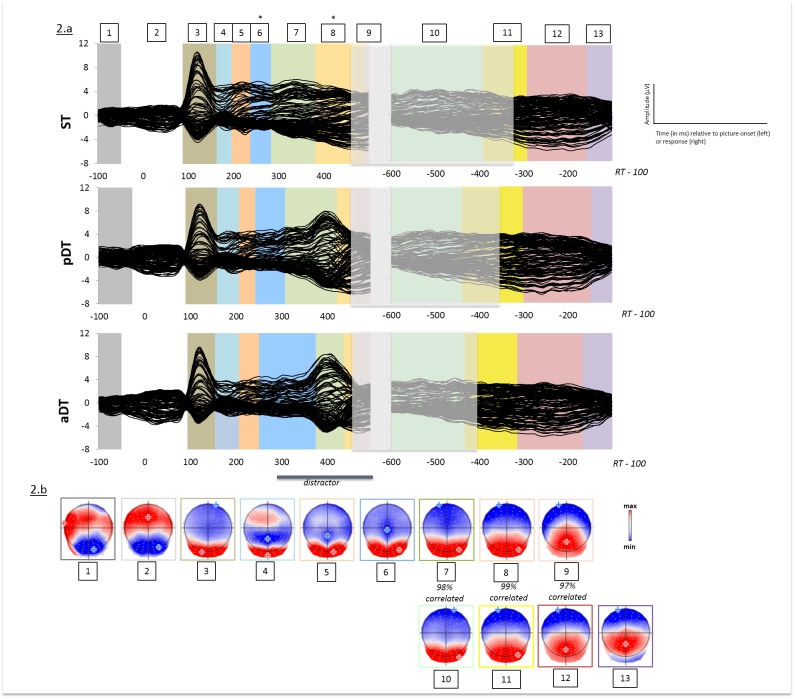
(a) Grand-average ERPs (128 electrodes) for each task (Single task ST, passive dual-task pDT and active dual-task aDT) when distractors were tones from 100 ms prior to picture onset to 100 ms before the verbal response and temporal distribution of the topographic patterns revealed by the spatio-temporal segmentation analysis. Stable electrophysiological configurations are color-coded. The white shaded area illustrates overlap between stimulus-aligned and response-aligned data as a function of grand-averaged production latencies for each task. (b) Map templates for the nine stable topographies observed from 100 ms prior to picture onset to RT-100. Positive (red) and negative (blue) values are displayed as well as maximal and minimal scalp field potentials. Correlations between map templates 7, 8, 9 and 10, 11 and 12 respectively are indicated. * indicate significant differences (p < 0.05) observed on any specific topographic pattern contrasting ST, pDT and aDT.

As microstates computed separately on stimulus-aligned and response-aligned data can correspond to the same topographic pattern, we performed correlations of visually similar microstates, which indicated that three out of the four map templates observed in the response-aligned data (maps 10, 11 and 12) correlated above 96% with the last three maps observed in the stimulus-aligned epochs (maps 7, 8 and 9 respectively) (see Fig 2B). This is likely to be due to overlap between stimulus-aligned and response-aligned epochs as a function of response times.

In order to better evaluate differences in duration across tasks, we applied a topographic fitting procedure (see Material and Methods) starting with the microstates that occurred in the time-windows displaying significant differences on waveform amplitudes on the stimulus-aligned data (i.e. from 190 ms post picture onset) to the end of the recorded ERPs in the response aligned data (see Fig 2).

Fig 2A plots the mean duration of all maps. A significant effect of Task on duration appeared on map 6 (p<0.007). The period of stable electrophysiological pattern at scalp labeled map 6, which displayed a posterior postivity and a negativity on central sites (see Fig 2B), started around 240 ms post picture onset. Planned comparisons indicated that the duration of map 6 was increased in aDT (mean duration: 124 ms) compared to ST (mean duration: 46 ms; p<0.03). Duration was marginally increased in aDT compared to pDT (mean duration: 64 ms; p<0.065) but the offset of map 6 was significantly delayed in aDT compared to pDT (p<0.007). Similarly, duration of map 6 was marginally increased in pDT relatively to ST (p<0.06) but offset was significantly delayed (p<0.006).

Significant differences on map duration were also observed on map 8 (p<0.0002) with increased duration in ST (mean duration = 110 ms) compared to pDT (mean duration = 28 ms; p<0.0004) and aDT (mean duration = 22 ms; p<0.0002). No differences were observed between the two dual-tasks. Statistics were not estimated for map 9 as its duration is constrained by the preceding map (here, map 8) and the end of the signal.

No significant differences were observed on the time-distribution of topographic patterns on response-aligned data.

#### Syllables–Waveform analysis

**Fig 3:**
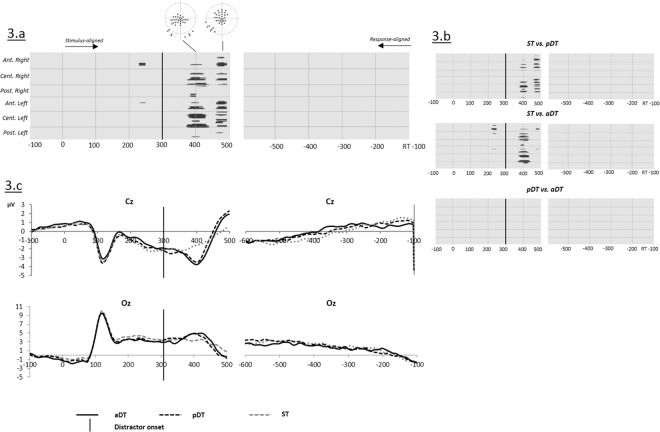
(a) Significant differences (ANOVA; p values) on ERP waveform amplitudes on each electrode (Y axis) and time point (X axis) between the three tasks (Single task ST, passive dual-task pDT and active dual-task aDT) when distractors used in dual-tasks were syllables. (b) Planned comparisons of significant differences on ERP waveform amplitudes on each electrode (Y axis) and time point (X axis). (c) Example of group averaged ERP waveforms for ST (dotted gray), pDT (dotted black) and aDT (solid black) on electrodes Cz and Oz. Vertical black solid lines indicate the onset of the distractors in dual-tasks.

Fig 3 shows the time points of significant amplitude differences from picture onset to 500 ms afterwards and on response-aligned ERPs as revealed by the ANOVA that contrasted the three tasks ST, pDT and aDT when the distractors were syllables.

A short effect (20 ms) was found on a cluster of 5 central electrodes around 220 ms post picture onset. More consistent differences were observed starting from 370 ms to 420 ms post picture onset and from 450 to 500 ms post picture onset on central and left occipital electrodes. These effects resulted from comparisons between ST, pDT and aDT (Fig 3B) with more negative waves on central electrodes observed for both dual-tasks relative to ST around 400 ms (see Fig 3C).

The significant differences on amplitudes starting from 370 ms are attributed to the presence of a positive peak in both pDT and aDT compared to ST on left occipital sites and its negative counterpart on fronto-central sites (Fig 3C). This peak appeared between 70 and 120 ms after the onset of the syllable, likely reflecting the N1-P2 complex for auditory processing.

Overall, planned comparisons did not reveal any significant differences on waveform amplitudes between the two dual-tasks. No significant difference was observed on response-aligned ERPs.

#### Syllables—Spatio-temporal analysis

**Fig 4:**
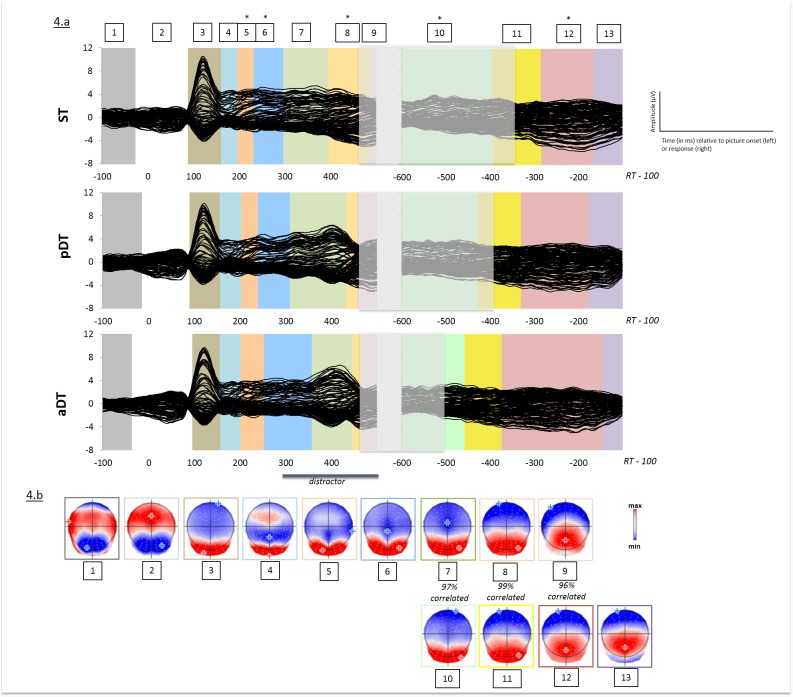
(a) Grand-average ERPs (128 electrodes) for each task (Single-task ST, passive dual-task pDT and active dual-task aDT) when distractors were syllables from 100 ms prior to picture onset to 100 ms before the verbal response and temporal distribution of the topographic patterns revealed by the spatio-temporal segmentation analysis. Stable electrophysiological configurations are color-coded. The white shaded area illustrates overlap between stimulus-aligned and response-aligned data as a function of grand-averaged production latencies for each task. (b) Map templates for the nine stable topographies observed from 100 ms prior to picture onset to RT-100. Positive (red) and negative (blue) values are displayed as well as maximal and minimal scalp field potentials. Correlations between map templates 7, 8, 9 and 10, 11 and 12 respectively are indicated. * indicate significant differences (p < 0.05) observed on any specific topographic pattern contrasting ST, pDT and aDT.

Similar to what was done for tones, a spatio-temporal segmentation analysis was run separately on the stimulus-aligned and the response-aligned grand-averaged ERPs of the three tasks. We also found 9 different periods of quasi-stable electrophysiological activity at scalp on the stimulus-aligned data and 4 topographic maps on the response-aligned data (see Fig 4). Note that these topographic maps were similar to those observed for tones and were observed in all conditions but with different time-distribution. Correlations of visually similar microstates also indicated that maps 10, 11 and 12 correlated above 96% with the last three maps observed in the stimulus-aligned epochs (maps 7, 8 and 9 respectively), likely due to overlap between stimulus-aligned and response-aligned epochs as a function of response times.

As for the tones conditions, the statistical analysis was restricted to the microstates that occurred from 190 ms post picture to the response-aligned data (see Fig 4).

Fig 4A plots the mean duration of all maps across tasks. In contrast to what was obtained for tones, additional significant differences were observed on response-aligned data (map 12).

We found a significant effect of Task on map 5 (p<0.0002) and map 6 (p<0.05) but also on map 8 (p<0.0002) on stimulus-aligned ERPs.

Similarly to map 6, map 5 displayed a central negativity and posterior positivity (Fig 4B) and started on average 200 ms after picture onset. Planned comparisons indicated that map 5 was significantly longer in the active dual-task aDT (mean duration = 52 ms) compared to the passive dual-task pDT (mean duration = 36 ms; p<0.006) and the single task ST (mean duration = 34 ms; p<0.0002). Duration of map 6 was also significantly increased in aDT (mean duration = 104 ms) compared to ST (mean duration = 66 ms; p<0.05) but it did not reach significance when compared to pDT (mean duration = 70 ms; p>0.1)

Planned comparisons were also computed for map 8. Increased duration of map 8 in ST (mean duration = 92 ms) compared to pDT (mean duration = 24 ms; p<0.0004) and aDT (mean duration = 20 ms; p<0.0002) was obtained. No differences were observed between the two dual-tasks. Statistics were not estimated for map 9 as its duration is constrained by the preceding map (here, map 8) and the end of the analyzed signal.

Note that interpretation of maps 8 and 9, which showed modulations across tasks in the stimulus-aligned data, would be limited given their properties: they characterized the very end of the stimulus-aligned signal and were highly correlated with maps observed on longer periods in the response-aligned data (maps 11 and 12). We will therefore rather rely on and interpret the response-aligned data for these microstates. Significant differences in duration were observed across tasks on map 12, in the response-aligned data, which started around 290 ms and 330 ms prior to vocal onset in the single task ST and the passive dual-task pDT respectively, and around 375 ms prior to vocal onset in the active dual task (see Fig 4A). Map 12, the duration of which was significantly increased in aDT (mean duration = 228 ms) compared to both pDT (mean duration = 158 ms; p = 0.025) and ST (mean duration = 122 ms; p<0.001), displayed a stronger negativity on anterior frontal sites. There was no difference between pDT and ST (p>0.1). Significant differences were also observed on map 10 (p<0.05), which were due to increased duration in ST (mean duration = 204 ms) relatively to aDT (mean duration = 140 ms; p<0.05) but not compared to pDT (mean duration = 170 ms). No other topographic pattern was affected by Task in the response-aligned data.

As a conclusion, qualitative comparison between the results obtained when distractors were tones or syllables show that the data can be summarized by the same pattern of quasi-stable electrophysiological activies.

Convergence was found between shifts of topographic maps on the one hand and increased production latencies on the other hand. Indeed, the results show that whereas during the dual-task with detection, only periods of topographic stability occurring between 200 and 300 ms post picture onset were extended when the distractor was a tone, additional microstates occurring in later time-windows were extended when the distractor was a syllable.

## Discussion

In this study, we examined the spatio-temporal dynamics of dual-tasks interference using EEG recordings, during picture naming tasks that varied as a function of the degree of attentional demand when auditory distractors involved in the concurrent tasks are verbal or non-verbal stimuli.

Behavioral results first show that both active and passive dual-tasks induced interference on picture naming as indicated by slowed down production latencies. Moreover, dual-tasks involving syllables induced larger interference on word production speed than those involving tones which led us to consider both distractors separately, as this could reflect different underlying interference mechanisms.

Our results showed that the mere perception of distractors (even when participants were instructed to ignore them; pDT) interfered to some extent with picture naming, and this interference seems magnified when attention was focused on distractors (aDT). Besides waveform differences between dual-tasks and picture naming in isolation around 400 ms post-stimulus onset (around 100 ms after auditory stimulus onset), the spatio-temporal ERP results revealed common modulations by dual-tasks of periods of stable electrophysiological activities starting around 200 ms, i.e. before the onset of the auditory stimulus and likely corresponding to lexical processes, for both verbal and non-verbal concurrent stimuli. An additional time-window of spatio-temporal modulations starting around 300 ms prior to vocal onset and associated to post-lexical processes, likely phonological-phonetic encoding, was only observed when verbal stimuli were involved in the dual-task.

Before focusing on the modulation of word planning processes by dual-tasks, we will discuss the dual-tasks interference *per se*.

### Dual-task interference

Several observations converge to associate the modulations of waveform amplitudes around 400 ms post-stimulus onset to the processing of the auditory stimulus *per se*. The time-period ranging from 380 to 420 ms post-stimulus onset likely corresponds to the N1 component that is often observed between 80 and 120 ms after auditory stimulus onset (here distractor onset; SOA = +300 ms) [49]. These effects were found whichever the distractor when comparing the dual-tasks with the single task, and were no longer present in planned comparison between the passive and the active dual-tasks. Therefore, the waveform differences around 400ms were likely related to the processing of an auditory stimulus on the one hand (aDT and pDT) and the absence of such a process in the other hand (ST).

Differences in amplitude of the auditory N1 component related to distractor processing, as a function of the degree of attention, would have indicated that the Task2 (attending auditory stimuli) is also affected by the dual-task [49]. Although there seems to be differences in ERPs of the two dual-tasks within this time period, they did not reach significance. In contrast, differences were seen for tones, in short time periods before and after the N1, which could reflect modulations of waveform amplitudes on components of auditory stimulus processing as previously reported in dual-task experiments while speaking [50,51]. In contrast to PRP paradigms that monitor the response in Task2 as a function of Task1, we observed that response in Task1 (picture naming) was affected by the co-occurrence of Task2. The slowing down of Task1 response implies, at least, that attention has been distributed over the two tasks, which is compatible with the capacity sharing hypothesis [52–54]. In the capacity sharing hypothesis, the central capacity processes are distributed over the two tasks which limits resources for each individual task thus decreasing performance in both tasks. In our study, the association of the go response to filler items in the detection task enabled us to compare picture naming in the three conditions. At the same time, it prevented us from determining to what extent the resolution of Task2 (tone/syllable detection) was affected by attentional demand and whether there was a postponement of Task2. One should notice that modulations of early word encoding stages were observed for the active task in which there was a non-verbal distractor, and prior to the appearance of the distractor (as early as 200 ms). This suggests that, even though participants were instructed to ignore tones in one case, their attention was still divided. This was likely linked to the dual-task rather than the physical presence of the distractor itself. Note that recent work on dual-task interference brought evidence for a more mixed model encompassing both parallel and serial (bottleneck) processing [55]. In their study, Marti and colleagues (2015) [55] were able to disentangle the neural signal relative to each of the two tasks performed concurrently in the magneto-encephalographic (MEG) signal. They highlighted that (early) processes of the two tasks can be performed in parallel but that at some point, they become mutually exclusive. This was supported by the shortening of Task1 and lengthening of Task2. In the following, we discuss how lengthening of specific microstates reported in the present study can be associated to interference of specific sub-processes of the picture naming.

### Modulations of specific word planning processes

As such the ERP modulations discussed earlier are not sufficient to infer on which word encoding processes are affected by the dual-tasks. The spatio-temporal segmentation was run to determine whether the differences on waveform amplitudes seen on a great number of electrodes were due to different underlying generators or to shifts in latencies of specific ERP microstates. This also provided information as to how the increased latencies in naming were distributed in the course of word production. The first observation is that the distribution of map templates corresponding to quasi-stable electrophysiological activities was the same across experimental tasks. This indicates that there is no additional or qualitatively different processing stage between naming in isolation, naming while ignoring distractors or naming during distractor detection. However, we found that specific stable electrophysiological activities were extended in naming during dual-tasks.

When distractors were tones, the ERP microstate that started around 240 ms after picture onset was significantly extended as evidenced by effects on duration and offset in both dual-tasks and even more in the active dual-task. Delays or extensions of specific processing stages are supposed to reflect how much the resolution of the concurrent task (tone detection) affects the picture naming [14]. Relatedly, it informs on whether the underlying word encoding stages are under attentional demand. As announced in the Introduction, the SOA of 300 ms for the presentation of the auditory stimuli was based on previous observations associating the time-interval ranging from 200 to 400 ms after picture onset to lexical retrieval and encoding [28]. Here, the N2/P2 component, previously associated with lexical selection [27,56,57] peaked around 250 ms in the simple naming task. Therefore, and coherently with previous reports [19,20,58], our results suggest that lexical selection at least is under attentional demand.

Interestingly, and besides effects on stimulus-aligned data, a specific microstate observed in the response-aligned data was extended in the picture naming during syllable detection only. Indeed, similarly to tones, stable electrophysiological activities observed between 200 and 300 ms after picture onset and likely associated with lexical selection were extended in the dual-tasks with syllable distractors. However, in contrast to what was observed to tones, the duration of the stable electrophysiological activity that started about 300 ms prior to vocal onset (map 12, ending around 140 ms before vocal onset) was increased as well. Several observations converge to the hypothesis that differences observed after 400 ms correspond to modulations occurring during word form encoding. Indeed, assuming a serial model of lexical access [2,4], as differences up to 380 ms after picture onset seem to relate to lexical selection [56,59,60] phonological processes should take place afterwards. Relatedly, manipulation of word age of acquisition (AoA) which is thought to tap into lexical-phonological encoding [28,61] was associated with ERPs modulations around 400 ms after picture presentation [25,40,62]. This suggests that lexical selection but also phonological word form encoding are under attentional demand. However, the fact that this later time-window was affected only when distractors were verbal auditory stimuli seems to imply that phonological encoding does not require the same attention as lexical selection, which is modulated whichever the nature of the auditory distractor. Yet, this later encoding time-window was not affected by the mere presentation of an auditory verbal distractor (it is not a pure picture-word interference effect) as (i) the interference was only observed in the active dual-task and (ii) the amount of slowing in the passive listening was similar for tones and syllables. The active dual-task with verbal stimuli seems to have a specific impact on word form encoding whereas passive listening to the same verbal stimuli and responding to non-verbal auditory stimuli do not.

The here-reported results, in particular qualitative differences observed for verbal and non-verbal distractors, tackle the issue of the automaticity of speech planning processes and how it might relate to cross-talk mechanisms.

### Attentional demand in word planning and cross-talk mechanisms

Dual-task interference on word production is thought to concern only response selection while perceptual processes and response execution (prelinguistic and postlexical stages in word production) would be spared [18]. Our results comfort the idea that lexical selection is under general attentional demand [6–10,13,19,20,58] and that post-lexical processes are more automatic, but the pattern depends on which kind of auditory stimuli the participants are asked to process in the concurrent tasks. When participants are asked to perform a tone detection task simultaneously to picture naming, phonological word form encoding is not modulated [19,20]. In contrast, when participants are asked to perform a syllable detection task, these later encoding processes seem to be affected. This suggests that whereas speakers can retrieve the phonological form of the word while responding to tones, they cannot do it with the same ease while responding to syllables. This observation could in fact indicate that the attention requirements of phonological encoding rely on a cross-talk mechanism.

The cross-talk hypothesis assumes a relation between response selection of Task1 and Task2 [22] and suggests that the amount of interference depends heavily on the content of the information processed [18], in other terms, on the degree of overlap between the tasks or the mental processes involved. The more similar the two tasks are in terms of specific processing needed, the more interference is observed [63]. It has already been claimed that the degree of interference induced by concurrent tasks is directly related to the degree of proximity of neural networks involved in the resolution of the tasks [64]. During phonological encoding, the phonological segments of each morpheme are selected and then combined into syllables. Thus, we can assume that the neural circuits used to activate/select the segmental content of the word were, at the same time, engaged in processing the distractors when they were syllables but not when they were tones. By contrast, qualitatively different neural circuits would be recruited to process the tones.

In line with the idea that two tasks requiring relatively independent brain networks should show decreased interference [64], weaker interference was observed for tones compared to syllables which display a specific impairment of the phonological word form encoding stage. In order to account for discrepancies across experiments in the capacity demand of phonological encoding, Cook & Meyer (2008) [20] argued that this encoding stage (and phoneme selection in particular) was indeed under attentional demand, but that demonstrations of that would be at variance with other processes such as speech monitoring. Our results reflect that attention requirements during word form encoding critically depend on special circumstances such as the degree of overlap between the constituents of distractors and of words (cross-talk). Whether the differences in the attention requirements of lexical selection and phonological encoding reflect respectively domain-general or domain-specific attention [65] remains to be clarified. Finally, this result speaks in favor of variability in capacity demands of subcomponents of word planning, which is not predicted by current models of word production.

It should be noted, however, that our results are contingent upon the SOA used of +300 ms which is thought to co-occur with lexical processes. Possibly, the encoding processes affected by the dual-task and the amount of interference may vary as a function of SOAs [13]. Note that different kinds of stimuli (i.e. verbal vs. non-verbal) might require different amounts of attention once they have been presented. Following this idea, one can speculate that attending to tones differs from attending to syllables and that presenting tones or syllables with a different timing relative to picture onset would lead to a different pattern of results. These issues offer precious perspectives to the present study and should be investigated in the future.

## Conclusions

In this study on dual-task interference on word production, we used ERPs and spatio-temporal analyses to demonstrate that lexical selection and word form encoding are under attentional demand, but are not affected to the same extent by verbal and non-verbal concurrent auditory stimuli. The differences reported for these two types of stimuli might reflect the sensitivity of some aspects of word production (e.g. phonological word form encoding) to cross-talk mechanisms.
